# Anion receptors containing thiazine-1,1-dioxide heterocycles as hydrogen bond donors

**DOI:** 10.3762/bjoc.6.50

**Published:** 2010-05-19

**Authors:** Hong-Bo Wang, James A Wisner, Michael C Jennings

**Affiliations:** 1 Department of Chemistry, University of Western Ontario, 1151 Richmond St., London, Ontario N6A 5B7, Canada

**Keywords:** anions, hydrogen bonds, receptors, sulfonamides, supramolecular chemistry

## Abstract

The synthesis, X-ray crystal structures and anion recognition properties of two receptors containing thiazine-1,1-dioxide heterocycles as hydrogen bond donating subunits are reported. The newly synthesized receptors display much different anion selectivities in acetone-*d*_6_ than *N*,*N*′-diphenyl-1,3-disulfonamidobenzene that was used as a comparison. The selectivity exhibited by one of the new receptors for chloride anions can be attributed to greater steric demand in the cleft formed, in part, by its terminal phenyl rings; an effect that is absent in the comparison receptor.

## Introduction

**Figure 1:**
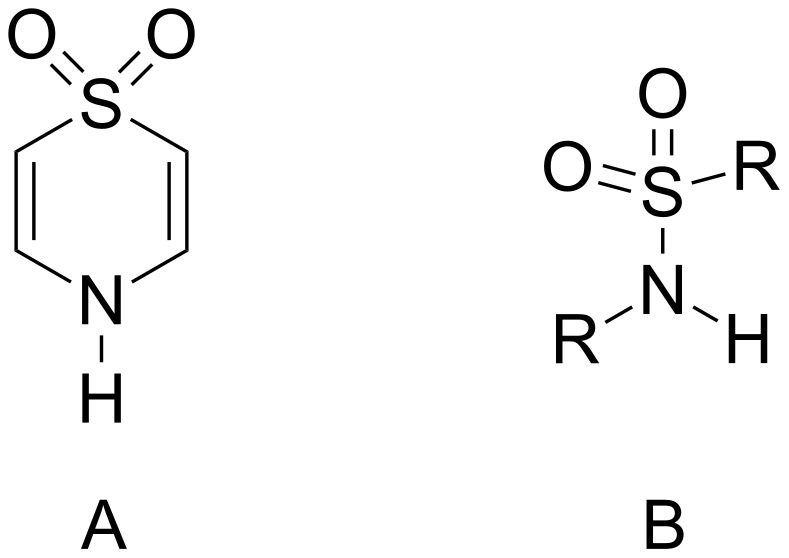
Structures of thiazine-1,1-dioxide heterocycle (A) and sulfonamide function (B).

The synthesis of neutral hosts and study of their anion recognition properties is an area of research that has grown in interest over the past several years owing to the potential use of such receptors in environmental, biomedical and materials applications [1–2]. The basic design methodology for these hosts has largely focused on the use of nitrogen-based hydrogen bond donor groups such as amides [3–4], ureas [5], pyrroles/indoles/carbazoles [6–7] and sulfonamides [8–23] to complex the anionic targets in a topologically complementary fashion. Sulfonamides are an interesting case as the hydrogen bond donor is often significantly more acidic (p*K*_a_ approx. 11 for simple *N*-phenylaryl sulfonamides such as **3** (see below)) than that presented by other groups typically incorporated in these frameworks. The greater acidity of such a subunit can be an advantage by providing greater potential hydrogen bond donor strength with anionic guests. Alternatively, the possibility of deprotonation in some specific systems by basic anions such as carboxylates or fluoride can be employed as an indicator for these species. Regardless, the incorporation of sulfonamide functional groups has typically been realized synthetically by sulfonylation of an amine to form a sulfonamide product. This approach is somewhat limited, from a design perspective, in that the majority of examples to date consist of sulfonamides derived from a few commercially available starting materials such as benzenesulfonyl, toluenesulfonyl, dansyl, and benzenedisulfonyl chlorides [8–23]. We have recently investigated thiazine-1,1-dioxide heterocycles (Figure 1A) as hydrogen bond donor groups in the formation of double helical complexes [24]. The parent heterocycle can be viewed as a cyclic, vinylogous sulfonamide that presents a different spatial, conformational and electronic relationship between the sulfonyl and NH subunits than that of a typical sulfonamide function (Figure 1B). It is a simple matter to access many such derivatives with this framework using straightforward synthetic methods and inexpensive materials and reagents. Herein, we describe an illustrative synthesis of two anion hosts incorporating these heterocycles and compare their binding affinities with some common anionic guests to that of an analogous benzene disulfonamide anion receptor.

## Results and Discussion

**Figure 2:**
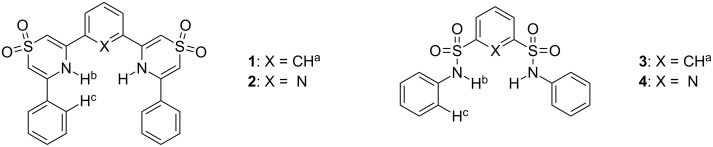
Structures of anion receptors **1**–**4**.

The two receptor structures **1** and **2** (Figure 2) were chosen with the intent of evaluating their efficacy in comparison to the known anion host *N*,*N*′-diphenyl-1,3-disulfonamidobenzene **3** (Figure 2) [21]. Originally investigated for anion recognition by Crabtree and coworkers, **3** was considered a representative comparator given the similar stereochemical arrangement of the two NH donors and the 1,3-benzenediyl spacer. The incorporation of a pyridyl spacer in **2** was chosen to examine the possible effect the ring nitrogen atom might have on the preorganization and anion recognition properties of the resulting host in comparison to **1**. It is well known that structurally related 2,6-dicarboxamidopyridine containing hosts have markedly different properties compared to their analogous isophthalamide derivatives in these regards as well [25–26]. It is an indication of the potential versatility of the synthetic method described here that the elusive 2,6-disulfonamidopyridine host **4** (Figure 2) that would provide a more direct comparison to **2** is at present unknown and likely synthesized only with some difficulty.

**Scheme 1:**
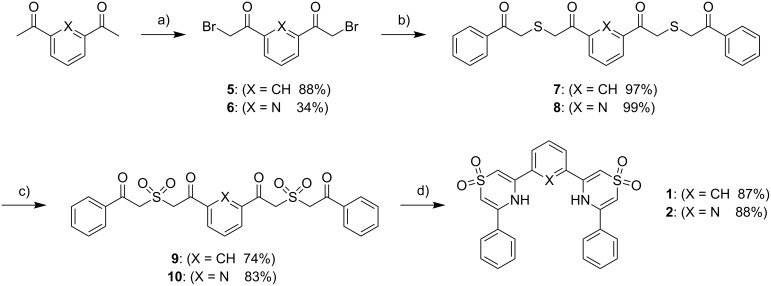
Syntheses of **1** and **2**. Reaction conditions: (a) (X = CH) NBS, TsOH, CH_3_CN, reflux or (X = N) Br_2_, AlCl_3_, Et_2_O, 0 °C; (b) 2,6-lutidine, α-mercaptoacetophenone (2 equiv), CH_2_Cl_2_; (c) UHP/TFAA, CH_3_CN; (d) NH_4_OAc, AcOH, reflux.

The syntheses of receptors **1** and **2** are summarized in Scheme 1. α,α′-Dibromo-1,3-diacetylbenzene (**5**) and α,α′-dibromo-2,6-diacetylpyridine (**6**) are both simply generated by bromination of the corresponding diacylarenes. The reaction of either dibromide with α-mercaptoacetophenone in the presence of 2,6-lutidine yields dithioether intermediates **7** and **8**. Oxidation of these dithioethers to the disulfones **9** and **10** with urea-hydrogen peroxide (UHP) and trifluoroacetic anhydride (TFAA) in acetonitrile at room temperature proceeds in high yields. The final products **1** and **2** were obtained by the cyclization and dehydration of these intermediate disulfones with ammonium acetate in refluxing glacial acetic acid. Overall, the yields of receptors **1** and **2** are 62% and 72% respectively, from the dibromides. The simplicity and mild nature of these transformations make them easily applicable to the derivatization of most α-bromoacyl functional groups should one desire the installation of this subunit in a potential host.

**Figure 3:**
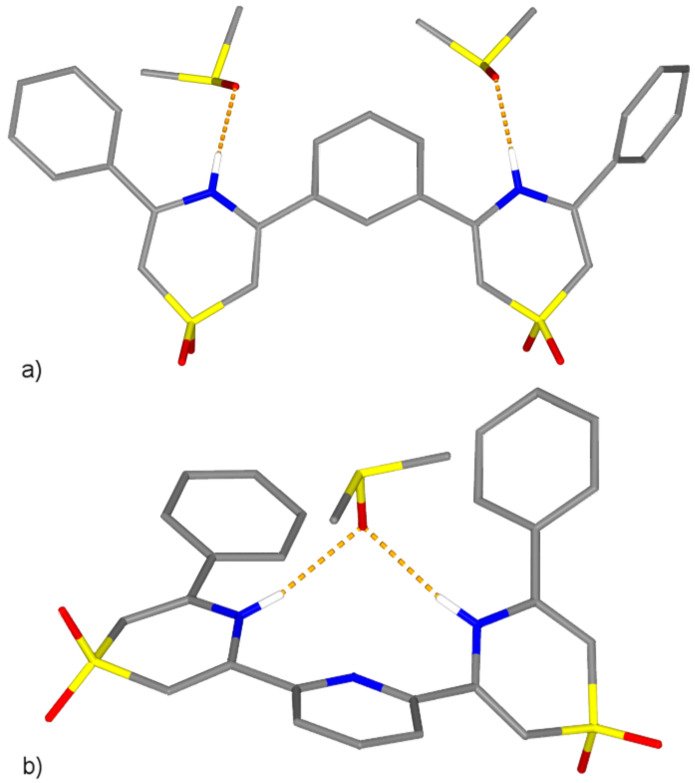
Stick representations of the X-ray crystal structures of (a) receptor **1** and (b) receptor **2**. Non-acidic hydrogen atoms omitted for clarity. Red = oxygen, blue = nitrogen, yellow = sulfur, grey = carbon. Hydrogen bonds denoted by dotted orange lines.

The solid-state structures of both newly synthesized receptors were confirmed by X-ray diffraction analysis of single crystals grown by the slow diffusion of isopropyl ether into concentrated DMSO solutions of each (Figure 3). Unfortunately, attempts to co-crystallize the receptors with anionic guests in a number of organic solvents were unsuccessful. The conformations of the receptors in the solid state are surprisingly different given the similarity in molecular structure; the two receptors differ only in the replacement of an aryl CH in **1** for N in **2**. The structure of **1** is in an extended, approximately *anti-anti* conformation [27] where each of the NH groups is hydrogen bonded to a different DMSO solvent molecule in the lattice (N···O = 2.821(3) and 2.770(3) Å). In contrast, **2** crystallizes in an approximately cleft-shaped *syn-syn* conformation where both NH groups are hydrogen bonded to a single DMSO solvent molecule (N···O = 2.971(2) and 2.950(3) Å). The difference may be rationalized by the presence of a weak intramolecular N-H···N hydrogen bond between the central pyridine ring and each of the two thiazine-1,1-dioxide rings in **2** that is necessarily absent in **1**. This conclusion is supported by the approximate 0.5 ppm chemical shift difference of the two receptor NH resonances in acetone-*d*_6_ (**2** > **1**) and similar shift differences observed in analogous hosts that are even larger when measured in the less competitive solvent CDCl_3_ [28].

The three receptors were each titrated in acetone-*d*_6_ with a number of TBA (tetrabutylammonium) salts of common anionic guests and the shifts of their ^1^H NMR resonances were observed as a function of anion concentration. In the majority of cases the downfield shift of the NH protons of the receptors was used to determine the stability constants. However, the addition of less than a half an equivalent of either acetate or dihydrogen phosphate anions to any of the three receptors resulted in the disappearance of the receptor NH proton resonances in their NMR spectra. In these cases, either the upfield shift of a CH resonance on the thiazine-1,1-dioxide rings (**1** and **2**) or the downfield shift of the 2-CH proton on the central phenyl ring of the receptor (**3**) was used to determine the stability constants. Non-linear least squares fitting of the data using the program EQNMR [29] yielded the complex stability constants in all cases. All of the titrations were fit to a 1:1 (receptor:anion) binding model except for the titration of receptor **1** with TBA chloride. In this case the data fit a 1:1 binding model that included a much weaker 2:1 (receptor:anion) component. It should also be noted that the data from titration of **3** with acetate had a binding constant that was too large to be reliably fit by this method. The titration results are summarized in Table 1.

**Table 1:** Stability constants (*K*_a_) determined by ^1^H NMR in acetone-*d*_6_ solution at 298 K for receptors **1**–**3** with a variety of anionic guests.

Anion^a^	Receptor **1**	Receptor **2**	Receptor **3**

Cl^−^	(2:1) 300 (1:1) 59000	300	4300
Br^−^	380	83	740
I^−^	53	—^b^	86
HSO_4_^−^	220	130	560
AcO^−^	12500	480	>10^5^
H_2_PO_4_^−^	540	360	79000

^a^Added as their tetrabutylammonium salts. Errors are estimated to be <10%. ^b^No change was observed in the ^1^H NMR of the receptor upon anion addition.

Receptor **1** exhibits a clear preference for a chloride anion guest over the other anions tested. Chloride is likely an excellent steric match to the cleft formed by the NH protons, the 2-CH proton of the central aryl ring and two of the *ortho*-protons of the terminal phenyl rings of **1** in a planar *syn-syn* binding conformation similar to that observed in the solid state structure of **2** with DMSO (Figure 1B). This conclusion is supported by the observation of significant downfield shifts of all three of these protons (Figure 2) in the ^1^H NMR spectrum upon chloride complexation (Δδ_max_ = 0.15 (H^a^), 1.91 (H^b^), 0.17 (H^c^) ppm). The progressively reduced affinity of **1** for bromide and iodide can be attributed both to their inability to fit into this idealized cleft conformation and their respectively decreasing efficacies as hydrogen bond acceptors. In fact, this observation is common to **1** and **2** and mirrors the behaviour of similar acyclic isophthalamide hosts studied previously by Crabtree and coworkers in CD_2_Cl_2_ [21]. Receptor **1** shows a preference for the complexation of chloride over acetate (5:1) and a distinct discrimination against the dihydrogen phosphate (>100:1 Cl:H_2_PO_4_) guest. Presumably, the larger size of the dihydrogen phosphate anion prevents complexation by **1** in a coplanar *syn-syn* conformation. The distortion of the receptor from a low energy coplanar binding geometry should reduce its affinity for such guests, despite their greater basicity. This supposition is supported in the case of dihydrogen phosphate by a very small Δδ_max_ for H^a^ observed when **1** is titrated with this anion (0.01 ppm) though Δδ_max_ of protons H^c^ remains significant (0.15 ppm) indicating their continued participation in the binding event. The titration of **1** with the less basic but similarly sized and shaped HSO_4_^−^ anion displays an *upfield* shift of H^a^ (Δδ_max_ = −0.05 ppm) and a reduced downfield shift of H^c^ (Δδ_max_ = 0.07 ppm).

Replacement of the central phenyl ring spacer of **1** for pyridine in **2** results in a significant reduction of the association constants for all of the anions tested. This result was expected as a consequence of repulsion of anionic guests by the lone pair of the pyridine ring nitrogen atom upon binding in the cleft of the receptor. The other outcome of this replacement is a loss of any selectivity for chloride and, in fact, a slight preference by receptor **2** for acetate and dihydrogen phosphate.

The simple disulfonamide receptor **3** exhibits very different complexation behaviour than **1** and **2** with the anions investigated. Receptor **3** displays a strong preference for acetate and dihydrogen phosphate over all of the other anions investigated in this solvent. The binding constant for chloride is reduced by an order of magnitude but is still preferred over bromide even though the affinity of **3** for the latter guest has approximately doubled in comparison to receptor **1**. No change in the ^1^H NMR spectrum of **3** is observed upon the addition of iodide. We believe that the differences in anion binding between these two receptors can be satisfactorily explained by the differences in their cleft geometries. The central three atoms (NH and CH^a^) that define the binding cleft in both **1** and **3** circumscribe a very similar meridian. In fact, we manipulated the single crystal molecular structures of **1** and the 4,4′-di-*t*-butyl derivative of **3** [30] (available from the Cambridge Crystallographic Database #1003/6124) by rotating the two relevant dihedral angles to bring the NH groups into plane with their central aryl rings in an idealized *syn-syn* conformation. Measurement of these “closest approach” N···N distances in the two models yields values of 4.76 and 4.77 Å for **1** and the derivative of **3**, respectively; a difference of 0.01 Å. The terminal phenyl rings of **3** do not, however, occlude this central cleft like those of **1** and **2**. Rather, they form a divergent “V”-shaped geometry upon chelation of anionic species by the two NH groups of **3**, regardless of whether coplanarity is maintained with the central benzene ring. The *ortho*-protons of these terminal rings (H^c^) are certainly too far away to contribute to the stability of the halide anion complexes that presumably form in this manner. The general result of this relaxation of the steric requirements for anion complexation by **3**, in comparison to **1**, is a marked increase in binding strength for all of the larger anions. Thus, the binding affinities of **3** for these larger anions follow the trend of their aqueous basicities (p*K*_a_ conj. acid): AcO^−^ (4.75), H_2_PO_4_^−^ (2.12), HSO_4_^−^ (−3), Br^−^ (−9), I^−^ (−10) [31].

## Conclusions

We have presented a simple synthetic route for the incorporation of thiazine-1,1-dioxide heterocycles as hydrogen bond donating subunits in two new acyclic anion receptors. The two new receptors **1** and **2**, were titrated with a number of anions and displayed very different complexation behaviour to the known disulfonamide receptor **3** that was used as a comparison. The difference can be attributed to the differing steric demands of the terminal phenyl rings in the two different receptor geometries despite the similar character of their central binding clefts. The steric effect of these rings in receptor **1** generates significant selectivity by the receptor for chloride versus the other, larger anions studied. The replacement of the central 1,3-benzenediyl spacer in **1** for 2,6-pyridinediyl in **2** greatly reduces the affinity of the resulting receptor for all of the anions examined and eliminates any selectivity for chloride. The synthetic approach described here can be easily adapted to the synthesis of oligomeric analogues of these two receptors that we expect will display an even greater selectivity for chloride anions and operate in more competitive solvent environments.

## Supporting Information

Details of synthetic procedures, characterization data for intermediates and final products, and binding isotherms for receptors **1** and **2** with TBA salts of anions.

File 1 Experimental details.

File 2 X-ray crystal data for receptor **1**.

File 3 X-ray crystal data for receptor **2**.
