# Supplementary material for: Anion receptors containing thiazine-1,1-dioxide heterocycles as hydrogen bond donors
Source: Beilstein J Org Chem. 2010 May 19;6:50. doi: 10.3762/bjoc.6.50 (PMC2887280; doi:10.3762/bjoc.6.50)
Supplement: File 1 — Experimental details. [file Beilstein_J_Org_Chem-06-50-s001.pdf]

Supporting Information

for

# Anion receptors containing thiazine-1,1-dioxide heterocycles as hydrogen bond donors

Hong-Bo Wang, James A. Wisner* and Michael C. Jennings

Address: Department of Chemistry, University of Western Ontario, 1151 Richmond St., London, Ontario, Canada, N6A 3K7

Email: James A. Wisner - jwisner@uwo.ca

*Corresponding author

## Experimental details

**General Methods.** Chemicals, including 1,3-diacetylbenzene and 2,6-diacetylpyridine, were purchased from Aldrich and used as received. All non-deuterated solvents were dried using an Innovative Technology solvent purification system SPS-400-5. All reactions were carried out under an atmosphere of dry $N_2$ unless otherwise stated. Chromatography was performed on Merck 240-400 mesh silica gel-60. $CDCl_3$ and acetone-$d_6$ were purchased from Cambridge Isotope Laboratories and dried over 3Å (acetone) or 4Å (chloroform) molecular sieves before use. $^1$H and $^{13}$C NMR spectra were collected on a Varian Mercury 400 MHz spectrometer. Spectra are reported with residual solvent peak as reference from TMS. Mass spectra were obtained on a Finnigan MAT 8200 mass spectrometer. $^1$H NMR titration experiments were performed on a Varian Inova 600 MHz spectrometer.

**Preparation of 5**

To a solution of 1,3-diacetylbenzene (1.667 g, 10.3 mmol) and *p*-toluenesulfonic acid monohydrate (5.871 g, 30.9 mmol) in acetonitrile (40 mL) was added *N*-bromosuccinimide (3.667 g, 20.6 mmol). The reaction was refluxed for 3.5 h. After the reaction mixture was cooled to room temperature, the solvent was evaporated. Dichloromethane (100 mL) and water (2×50 mL) were added to the residue, the layers separated and the organic layer dried over anhydrous $MgSO_4$. After evaporation of the solvent under reduced pressure, the white residue was α,α'-dibromo-1,3-diacetylbenzene, 88% yield. $^1$H NMR (400 MHz, $CDCl_3$) δ ppm 8.58 (s, 1H), 8.24 (dd, $J$ = 1.8, 0.59 Hz, 1H), 8.22 (dd, $J$ = 1.8, 0.59 Hz, 1H), 7.67 (t, $J$ = 7.82 Hz, 1H), 4.48 (s, 4H); $^{13}$C NMR (100 MHz, $CDCl_3$) δ 190.4, 134.5, 133.8, 129.6, 129.2, 30.3; HRMS (m/z) calculated for $C_{10}H_8Br_2O_2$ 319.9686, found 319.9683.

**Preparation of 6**

2,6-Diacetylpyridine (1.63 g, 10 mmol) was dissolved in dry ethyl ether (20 mL) and dry 1,4-dioxane (15 mL) at 0 °C under a nitrogen atmosphere. $AlCl_3$ (0.0133 g, 1 mmol) was added. Liquid bromine (20 mmol, 1.05 mL) in 1,4-dioxane (10 mL) was added dropwise via syringe over an h. The reaction mixture was stirred overnight. Then the solution was extracted with dichloromethane (20 mL) and washed with water (3×10 mL), 1M $NaHCO_3$ (10 mL), and dried over anhydrous $Na_2SO_4$. The solvent was removed under reduced pressure. After recrystallization from isopropanol, the pure product was obtained as a brown solid, 34% yield. $^1H$ NMR (400 MHz, $CDCl_3$) δ ppm 8.32 (d, $J$ = 7.8 Hz, 2H), 8.10 (dd, $J$ = 8.2, 8.0 Hz, 1 H), 4.82 (s, 4 H); $^{13}C$ NMR (100 MHz, $CDCl_3$) δ ppm 191.4, 150.4, 139.0, 126.6, 30.9; HRMS (m/z) calculated for $C_9H_7Br_2NO_2$ 320.9802, found 320.9727.

**General preparation of sulfides 7 and 8**

2-Mercapto-1-phenylethanone (13.8 mmol, 2.1g) and α,α'-dibromo-1,3-diacetylbenzene (or α,α'-dibromo-2,6-diacetylpyridine) (6.9 mmol, 2.21g) were dissolved separately in anhydrous dichloromethane (50 mL). The solutions were then purged with nitrogen for 3 min. The dichloromethane solution of 2-mercapto-1-phenylethanone was added via syringe. 2,6-Lutidine was added (10 mL) and the reaction mixture stirred for 16 h at room temperature. The reaction solution was extracted with dichloromethane (50 mL), and washed with 1 M citric acid (3×50 mL). The organic layer was dried over $MgSO_4$. After evaporation of solvent, the pure product was obtained.

**7**: light yellow solid, 97% yield. $^{1}$H NMR (400 MHz, $CDCl_3$) δ ppm 8.55–8.58 (m, 1 H), 8.18 (dd, *J* = 7.7, 1.7 Hz, 2 H), 7.96 (dd, *J* = 8.3, 1.1 Hz, 4 H), 7.55–7.63 (m, 3 H), 7.47 (t, *J* = 7.6 Hz, 4 H), 4.01 (s, 4 H), 3.99 (s, 4 H); $^{13}$C NMR (100 MHz, $CDCl_3$) δ ppm 194.0, 193.2, 135.8, 135.3, 133.6, 133.2, 129.3, 128.7, 128.6, 37.6, 37.45; HRMS (m/z) calculated for $C_{26}H_{22}O_4S_2$ 462.0960, found 462.0962.

**8**: light yellow solid, 99% yield. $^{1}$H NMR (400 MHz, $CDCl_3$) δ ppm 8.28 (d, *J* = 7.4 Hz, 2 H), 8.05 (t, *J*= 7.4 Hz, 1 H), 7.94–7.98 (m, 4 H), 7.56–7.61 (m, 2 H), 7.47 (t, *J* = 7.8 Hz, 4 H), 4.19 (s, 4 H), 4.00 (s, 4 H); $^{13}$C NMR (100 MHz, $CDCl_3$) δ ppm 194.1, 194.0, 151.3, 138.5, 133.5, 128.7, 128.6, 126.0, 38.0, 35.9; HRMS (m/z) calculated for $C_{25}H_{23}NO_4S_2$ 463.0913, found 463.0903.

**General preparation of sulfones 9 and 10**

Urea-hydrogen peroxide (UHP, 23.2 mmol, 2.28g) was introduced to a solution of trifluoroacetic anhydride (17.4 mmol, 3.65g) in acetonitrile (35 mL) and stirred for 3 min. The starting sulfide (2.9 mmol, 1.34g) was added slowly as a solid. The reaction mixture was then stirred for 1 h. Water (80 mL) was added to the reaction solution to precipitate a white residue that was filtered, washed with water and air dried.

**9**: white solid, 74% yield. $^{1}$H NMR (400 MHz, DMSO-$d_6$) δ ppm 8.66 (s, 1 H), 8.37 (dd, *J* = 7.8, 1.6 Hz, 2 H), 8.07 (d, *J* = 7.4 Hz, 4 H), 7.81 (t, *J* = 7.8 Hz, 1 H), 7.72 (t, *J* = 7.4 Hz, 2 H), 7.59 (t, *J* = 7.8 7.6 Hz, 4 H), 5.42 (s, 4 H), 5.32 (s, 4 H); $^{13}$C NMR (100 MHz, DMSO-$d_6$) δ ppm 189.7, 189.3, 136.2, 135.8, 134.4, 134.2,129.1, 128.9,

60.6; HRMS (m/z) calculated for [m + $H^+$] $C_{26}H_{24}O_8S_2^+$ 527.0834, found 527.0830.

**10**: white solid, 83% yield. $^1$H NMR (400 MHz, acetone-$d_6$) δ ppm 8.36–8.44 (m, *J* = 3.1, 9.8 Hz, 3 H), 8.11–8.16 (m, *J* = 8.0 Hz, 4 H), 7.69–7.75 (m, *J* = 8.8, 7.2 Hz, 2 H), 7.55–7.62 (m, *J* = 8.4, 7.8 Hz, 4 H), 5.59 (s, 4 H), 5.22 (s, 4 H); $^{13}$C NMR (100 MHz, DMSO-$d_6$) δ ppm 189.7, 151.0, 140.1, 135.8, 134.4, 129.1, 128.8, 126.4, 60.8, 59.5; HRMS (m/z) calculated for $C_{25}H_{21}NO_8S_2$ 528.1114, found 528.1176.

**General preparation of 1,4-thiazine-1,1-dioxides**

A mixture of sulfone (0.6 mmol, 0.316g) and ammonium acetate (6 mmol, 0.46g) in glacial acetic acid (20 mL) was heated under reflux 4.5 h and then cooled. The precipitated solid was filtered, washed with water and air dried.

**1**: light brown solid, 87% yield. $^1$H NMR (400 MHz, DMSO-$d_6$) δ ppm 10.65 (s, 2 H), 8.10 (s, 1 H), 7.90 – 7.92 (dd, *J* = 7.6, 1.2 Hz, 2 H), 7.77–7.79 (m, 4 H), 7.66 (t, *J* = 7.8 Hz, 1 H), 7.56–7.58 (m, 6 H), 6.69 (d, *J* = 3.7 Hz, 2 H), 6.42 (d, *J* = 3.7 Hz, 2 H); $^{13}$C NMR (100 MHz, DMSO-$d_6$) δ ppm 144.4, 143.3, 133.6, 133.4, 130.6, 129.8, 128.7, 128.0, 102.1, 101.7; HRMS (m/z) calculated for [m + $H^+$] $C_{26}H_{22}N_2O_4S_2^+$ 489.0944, found 489.0930.

**2**: light brown solid, 88% yield. $^1$H NMR (400 MHz, DMSO-$d_6$) δ ppm 10.72 (s, 2 H), 8.29 – 9.31 (d, *J* = 7.6 Hz, 2 H), 8.19–8.23 (dd, *J* = 7.2, 7.2 Hz, 1 H), 7.74 (d, *J* = 7.4 Hz, 4 H), 7.58–7.62 (t, *J* = 7.42 Hz, 2 H), 7.48–7.51 (t, *J* = 7.4 Hz, 4 H), 7.28 (d, *J* = 3.9 Hz, 2 H), 6.49 (d, *J* = 3.7 Hz, 2 H); $^{13}$C NMR (100 MHz, DMSO-$d_6$) δ ppm 148.5, 143.9, 14.0, 139.5, 133.6, 130.5, 128.9, 127.6, 123.2, 102.6, 101.9; HRMS (m/z) calculated for [m + $H^+$] $C_{25}H_{20}N_3O_4S_2^+$ 490.0922, found 490.0911.

**$^1$H NMR Titrations.** A 2 mL solution of anion receptor (typically 1–2.5 mM) was prepared in acetone-$d_6$. A portion (0.75 mL) of this solution was removed and the $^1$H NMR spectrum was recorded as the starting point for the titration. In 1 mL of the remaining anion receptor solution the tetrabutylammonium anion salt was dissolved at a concentration of 10–25 mM (i.e. 10 x the host concentration). Aliquots of guest solution were added successively to the NMR tube containing the host solution. The tube was shaken and the $^1$H NMR spectra were recorded after each addition.

**Binding isotherms for receptors 1–3 with TBA salts of anions.**

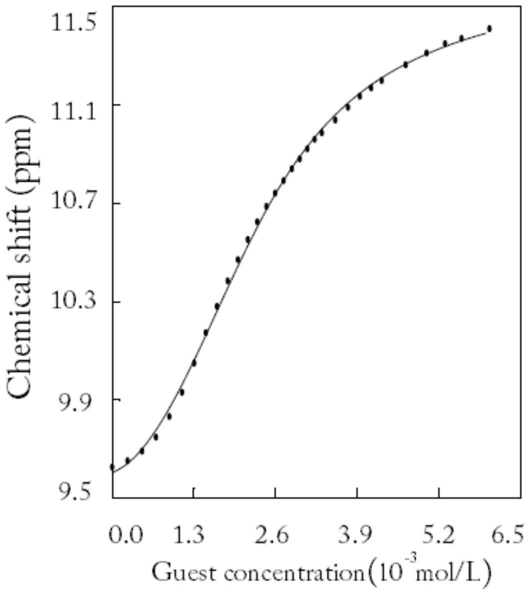


**Figure 1: The titration curve of anion receptor 1 with tetrabutylammonium chloride.**

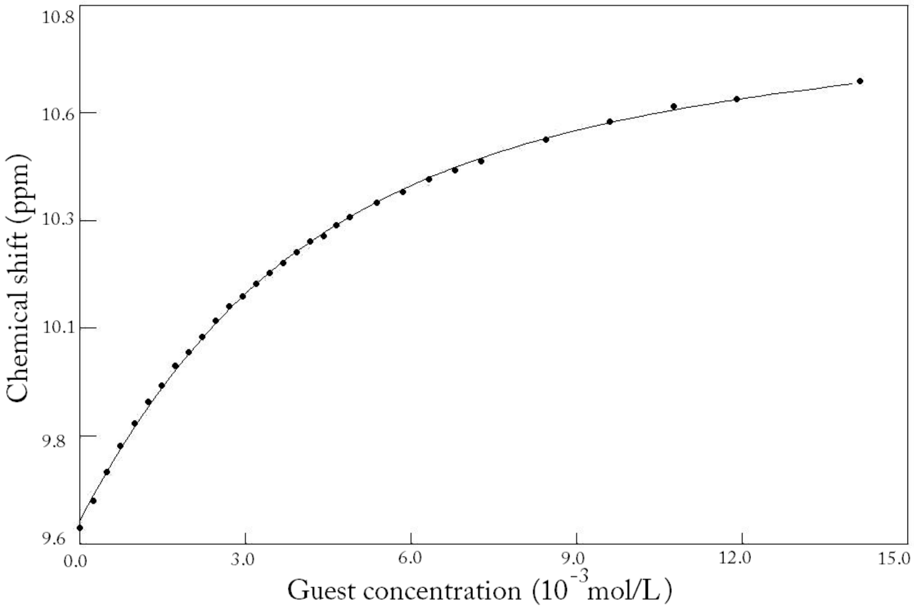


**Figure 2: The titration curve of anion receptor 1 with tetrabutylammonium bromide.**

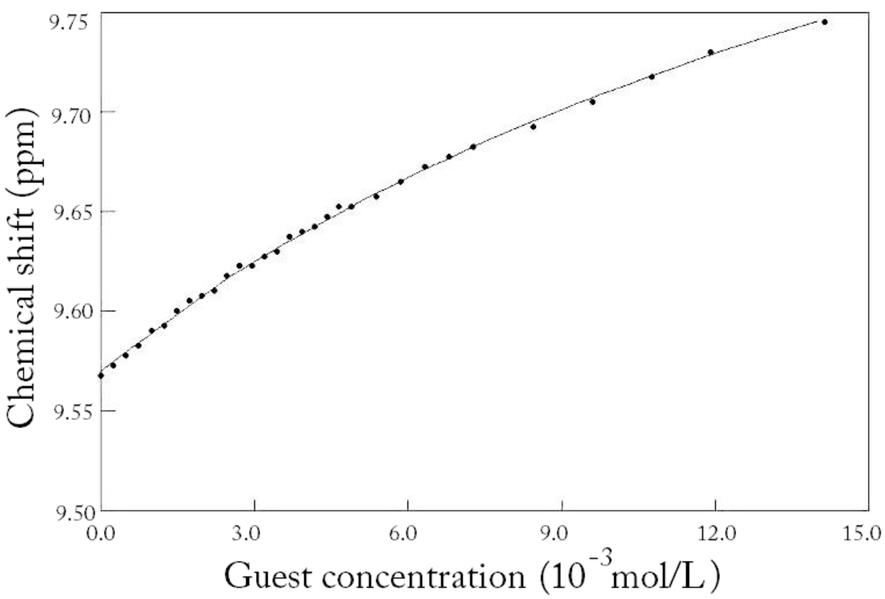

**Figure 3: The titration curve of anion receptor 1 with tetrabutylammonium iodide.**

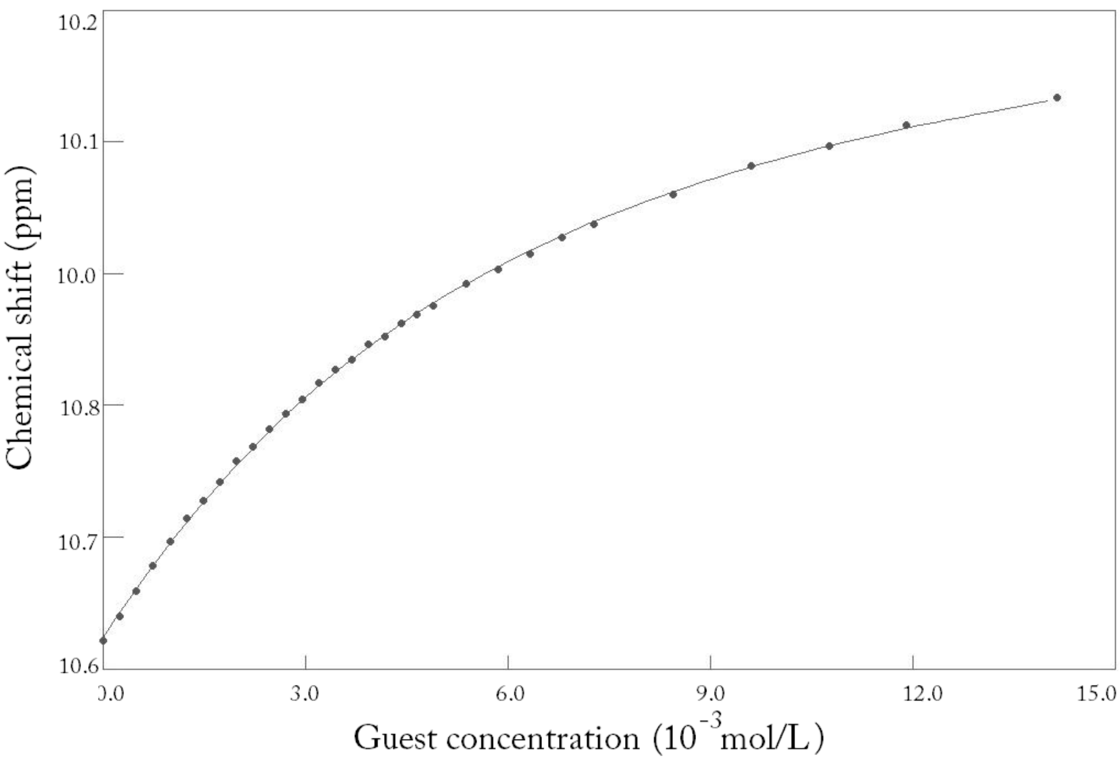

**Figure 4: The titration curve of anion receptor 1 with tetrabutylammonium hydrogensulfate.**

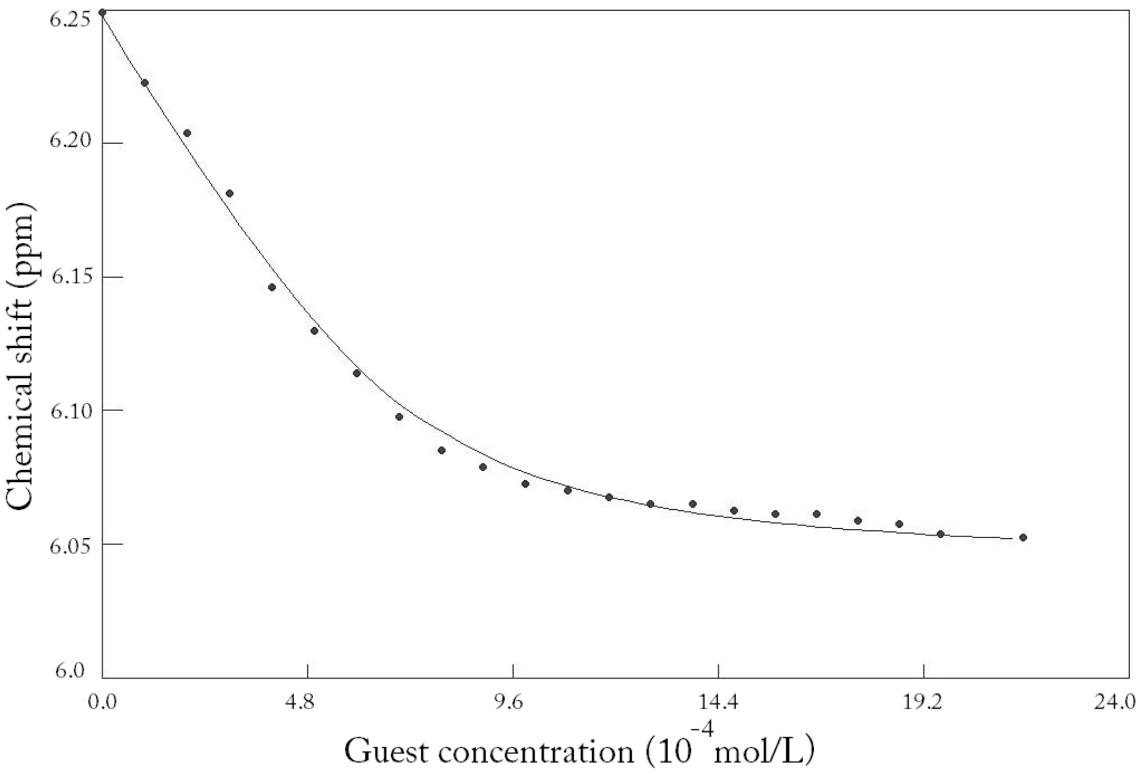


**Figure 5: The titration curve of anion receptor 1 with tetrabutylammonium acetate.**

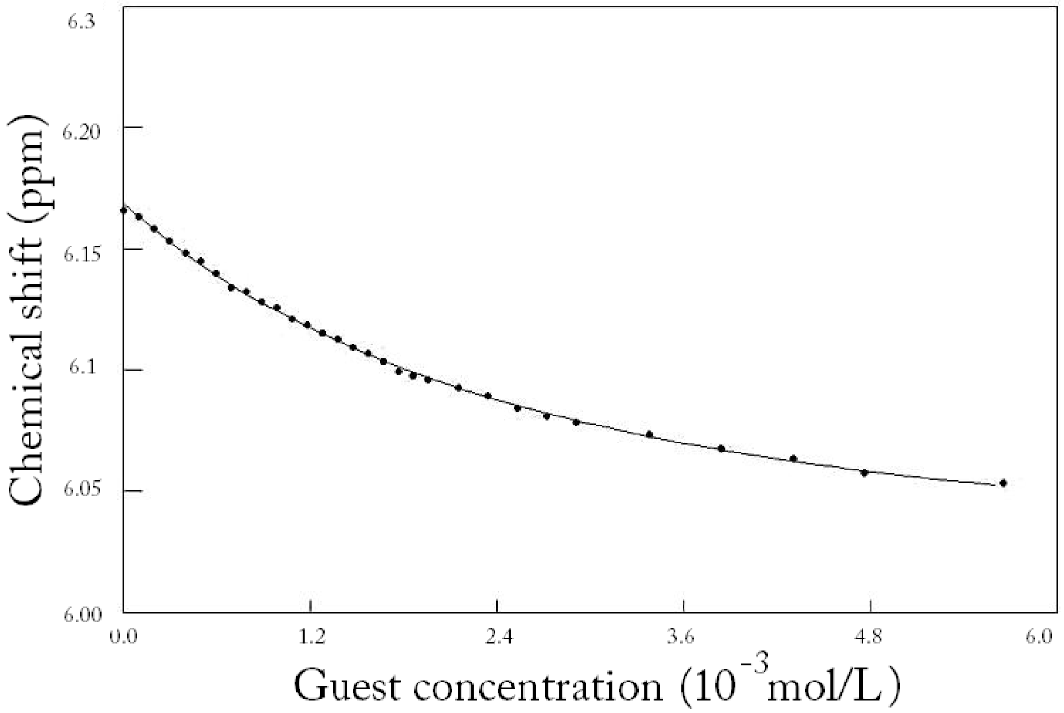


**Figure 6: The titration curve of anion receptor 1 with tetrabutylammonium dihydrogenphosphate.**

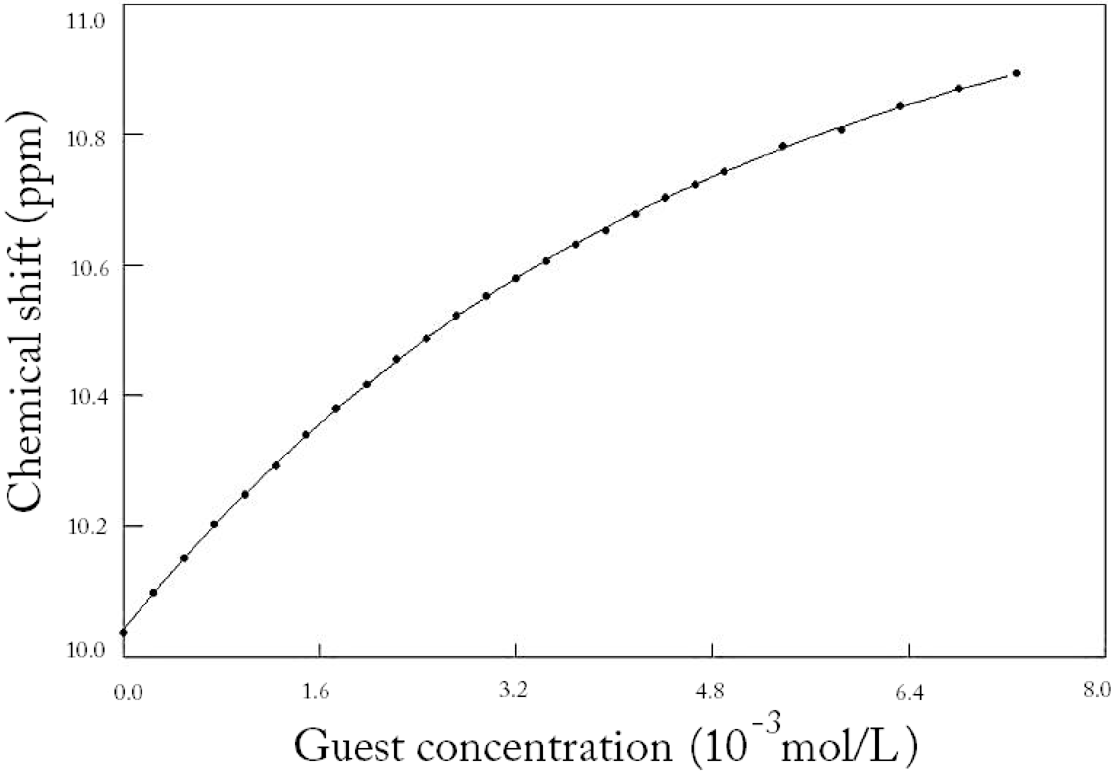


**Figure 7: The titration curve of anion receptor 2 with tetrabutylammonium chloride.**

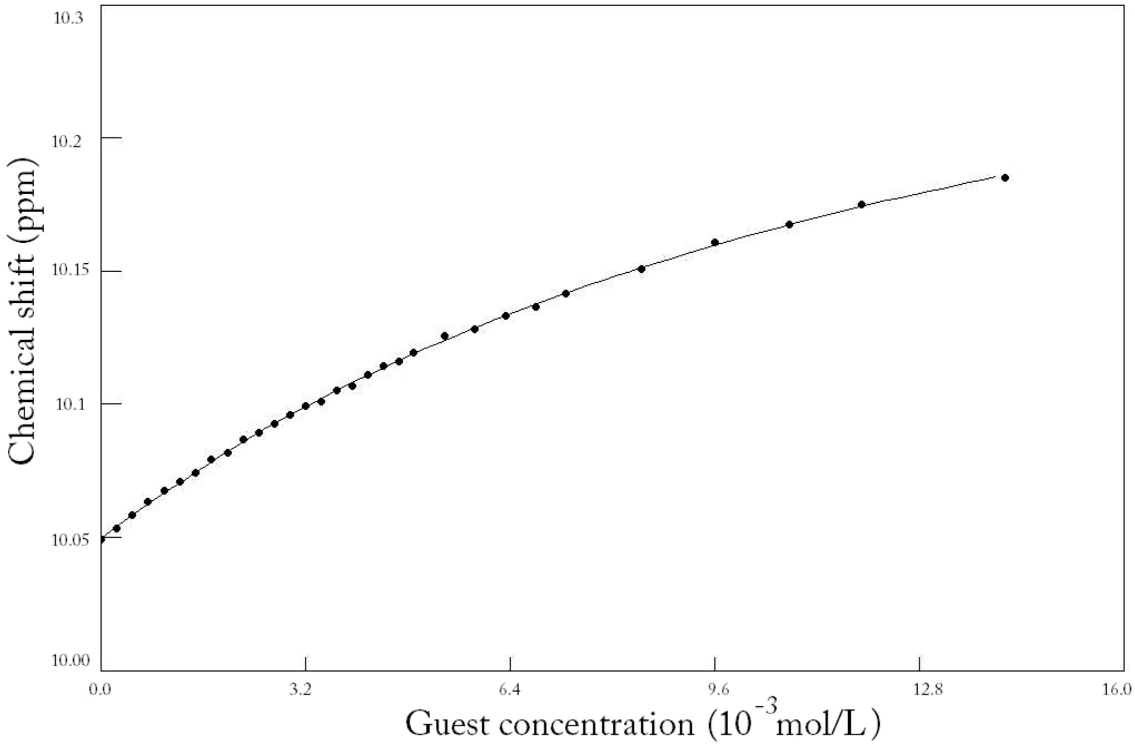


**Figure 8: The titration curve of anion receptor 2 with tetrabutylammonium bromide.**

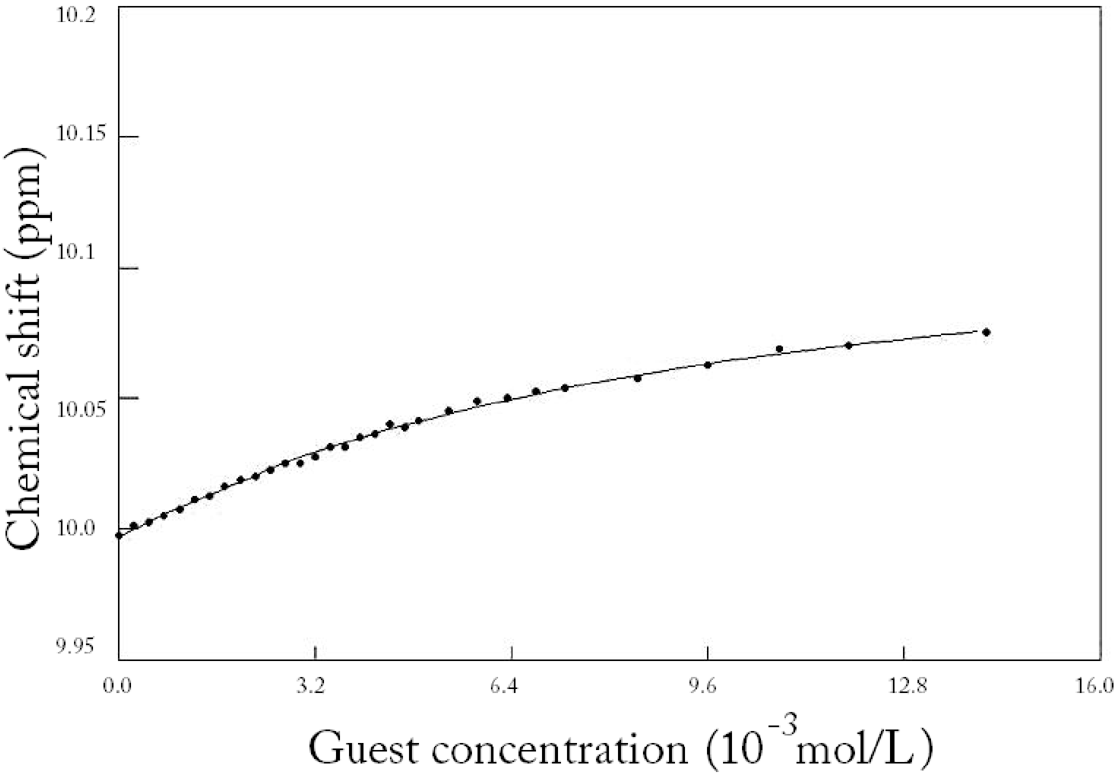


**Figure 9: The titration curve of anion receptor 2 with tetrabutylammonium hydrogensulfate.**

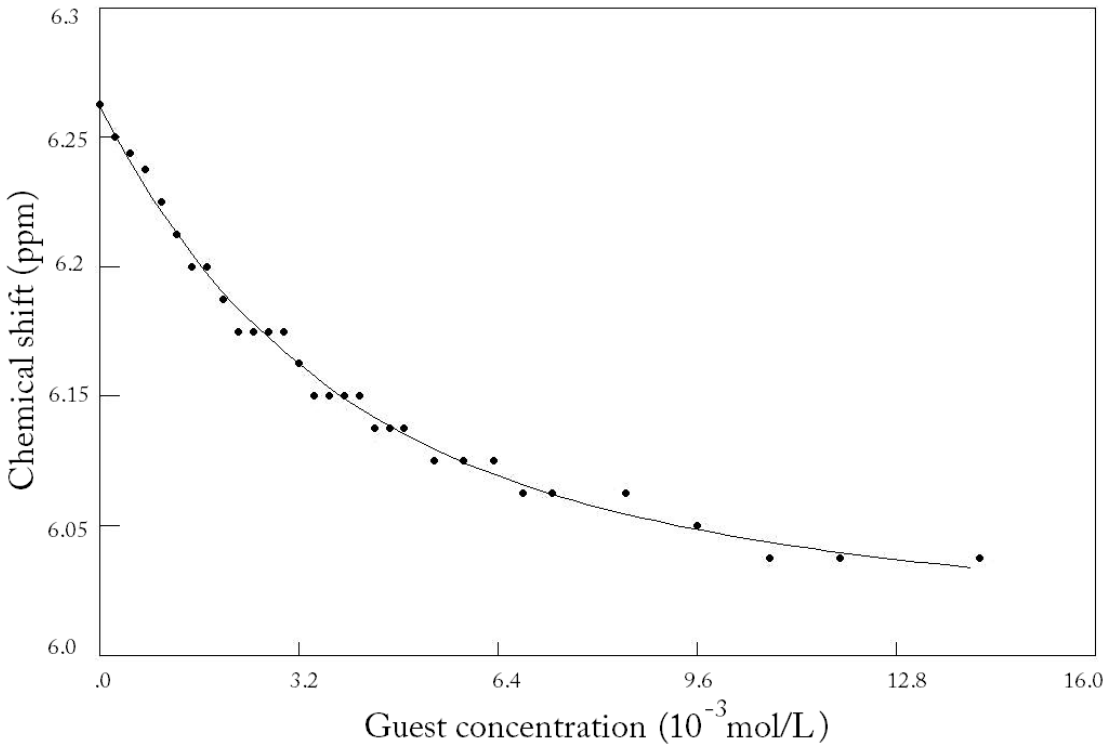


**Figure 10: The titration curve of anion receptor 2 with tetrabutylammonium acetate.**

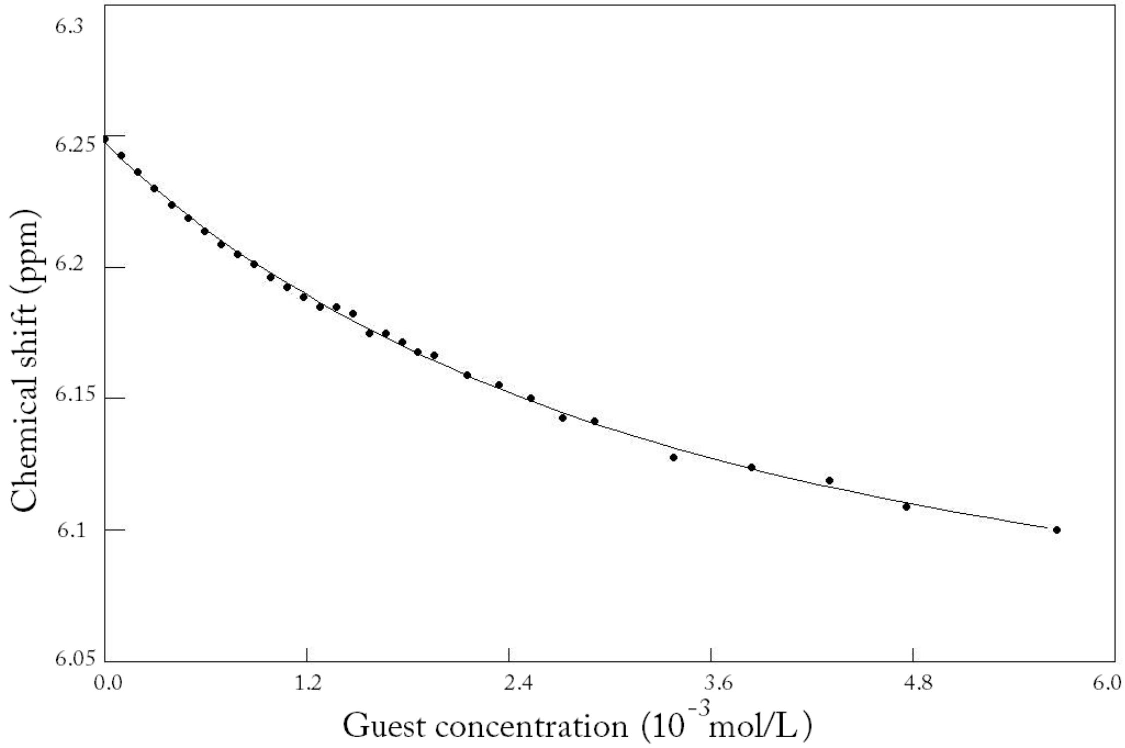


**Figure 11: The titration curve of anion receptor 2 with tetrabutylammonium dihydrogenphosphate.**

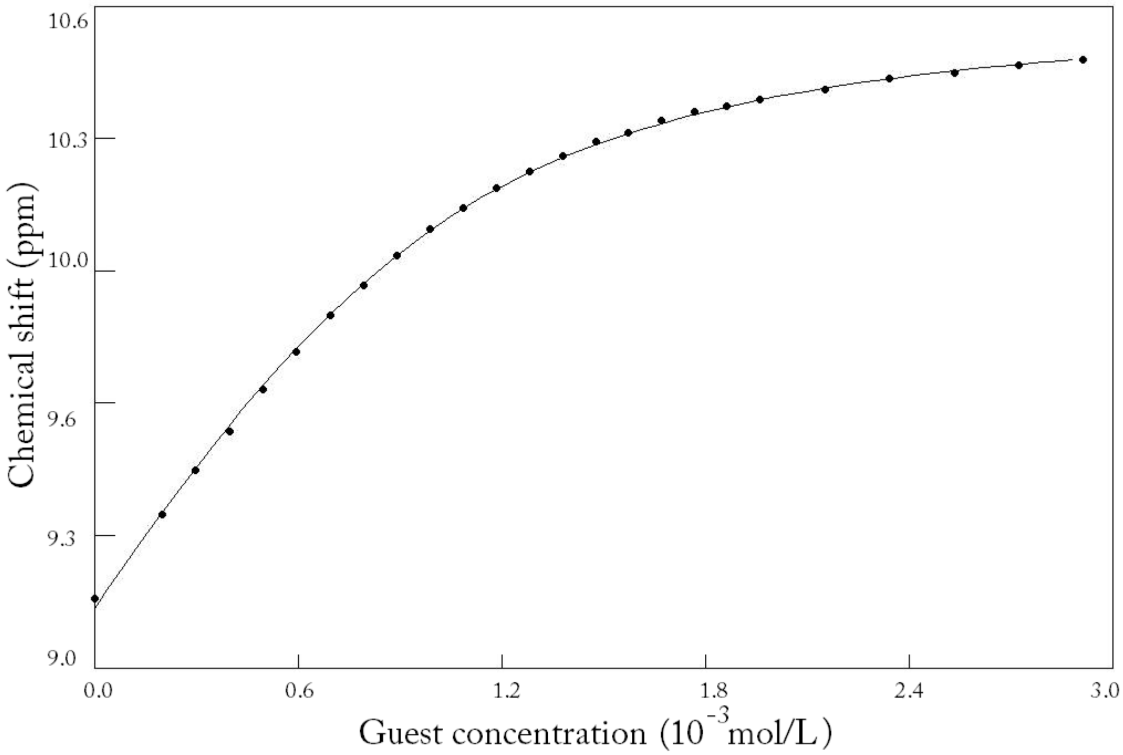


**Figure 12: The titration curve of anion receptor 3 with tetrabutylammonium chloride.**

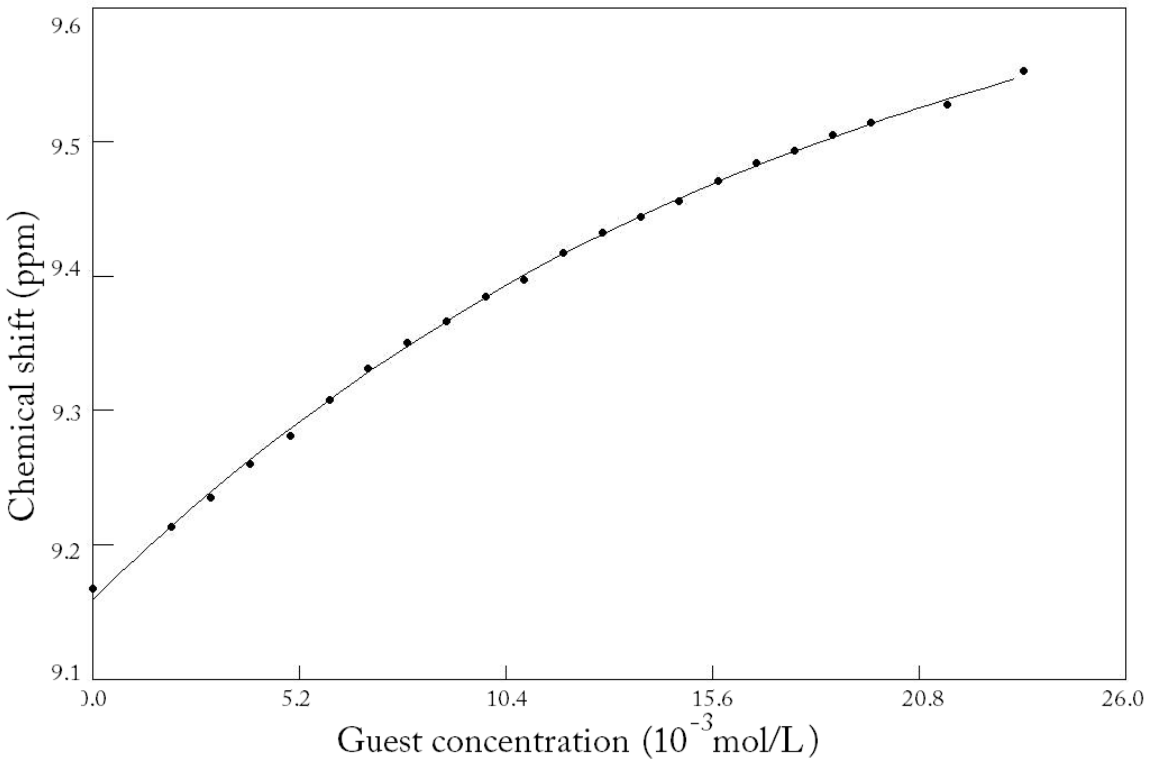


**Figure 13: The titration curve of anion receptor 3 with tetrabutylammonium bromide.**

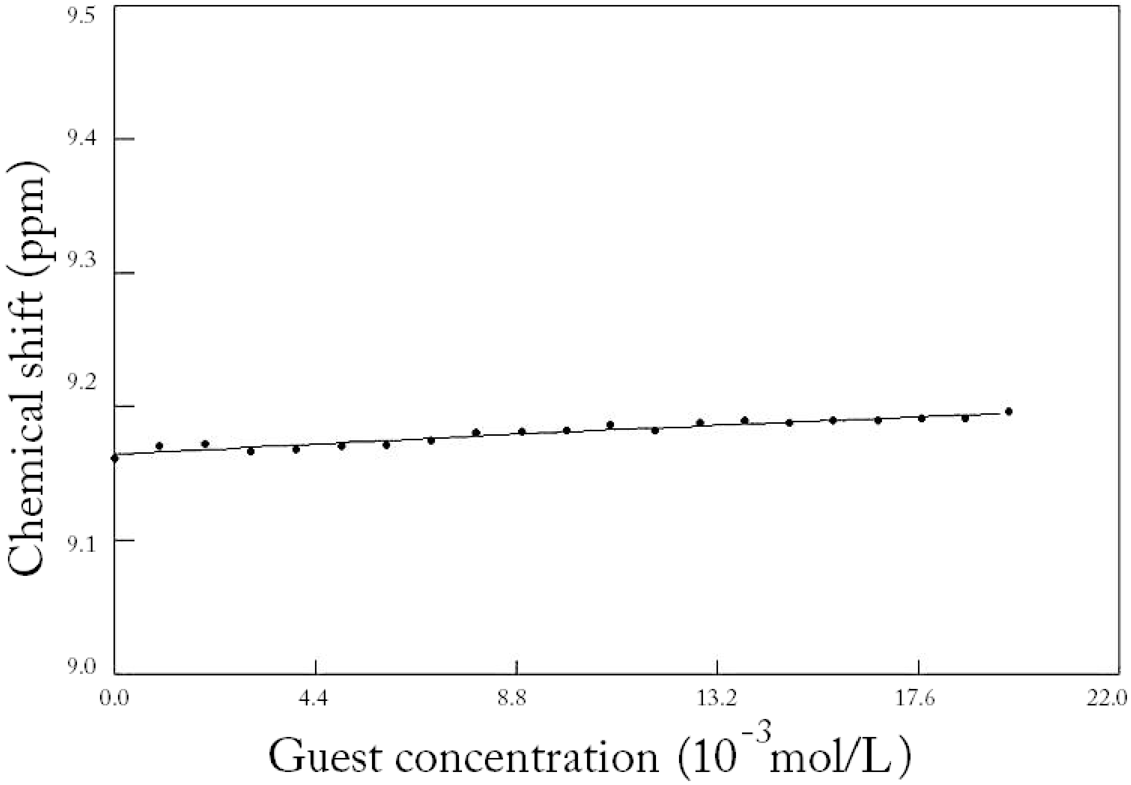


**Figure 14: The titration curve of anion receptor 3 with tetrabutylammonium iodide.**

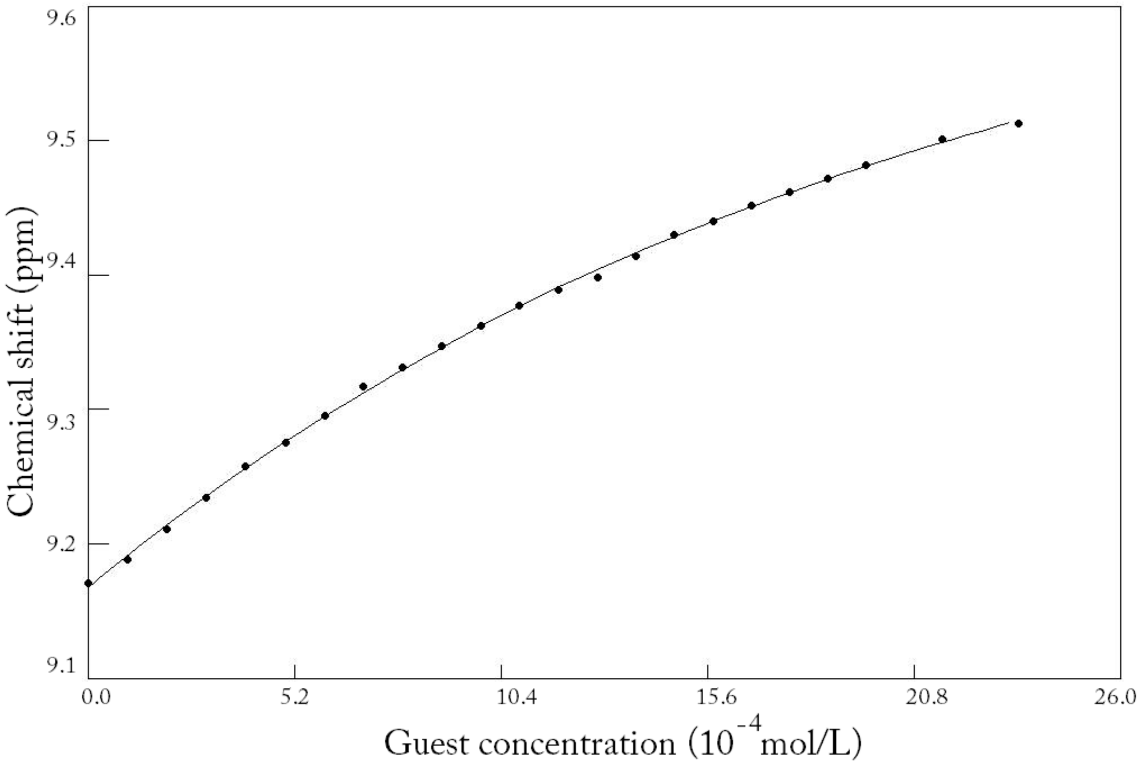


**Figure 15: The titration curve of anion receptor 3 with tetrabutylammonium hydrogensulfate.**

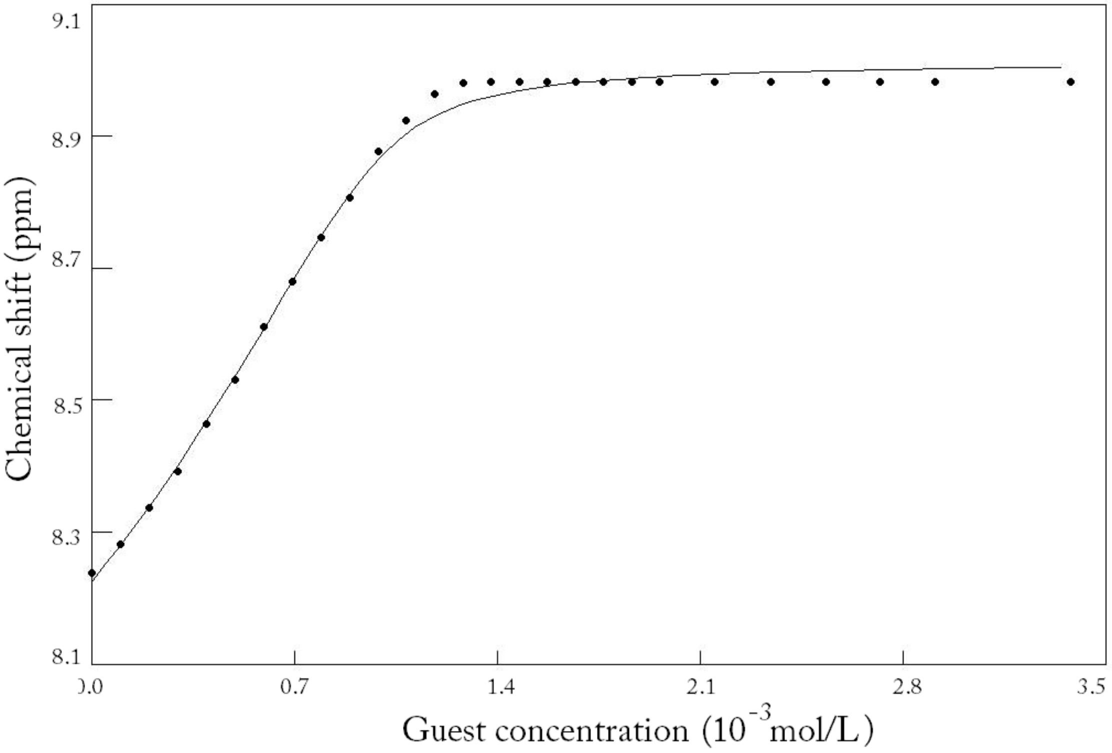


**Figure 16: The titration curve of anion receptor 3 with tetrabutylammonium acetate.**

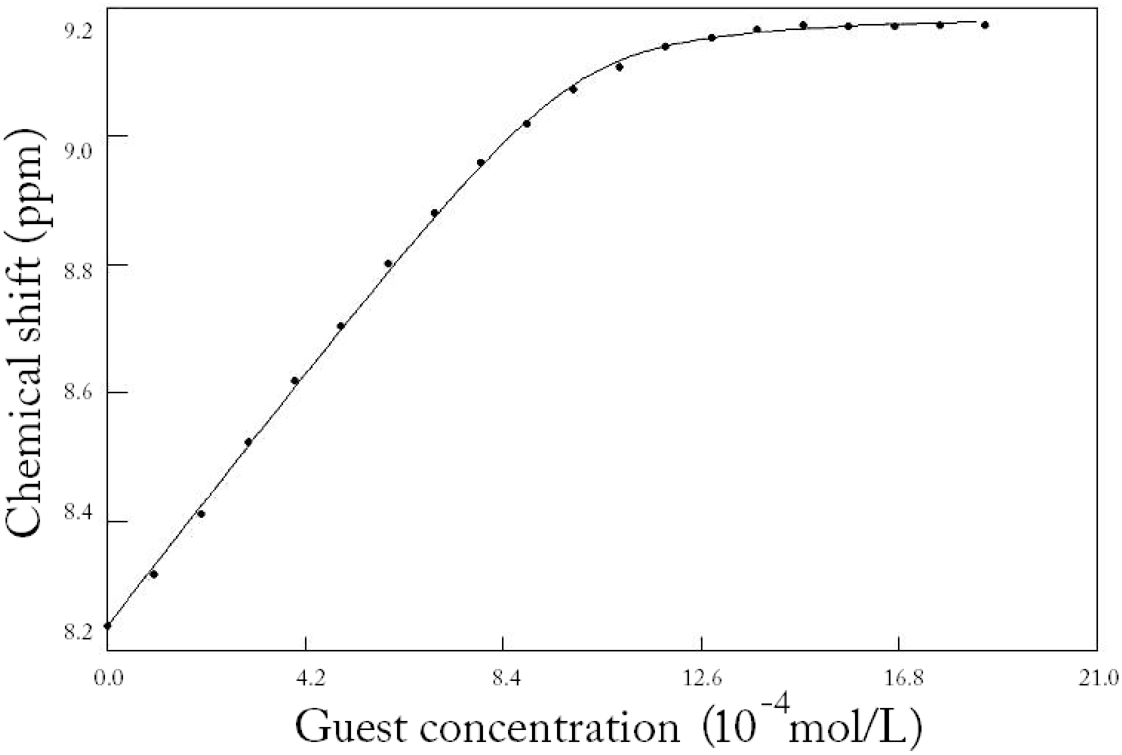


**Figure 17: The titration curve of anion receptor 3 with tetrabutylammonium dihydrogenphosphate.**
